# Hypoxia as a medicine

**DOI:** 10.1126/scitranslmed.adr4049

**Published:** 2025-01-22

**Authors:** Robert S. Rogers, Vamsi K. Mootha

**Affiliations:** 1 Department of Molecular Biology, Massachusetts General Hospital, Boston, Massachusetts, 02114, USA; 2 Broad Institute, Cambridge, Massachusetts, 02142, USA; 3 Department of Systems Biology, Harvard Medical School, Boston, Massachusetts, 02115, USA; 4 Howard Hughes Medical Institute, Boston, Massachusetts, 02115, USA

## Abstract

Oxygen is essential for human life, yet a growing body of research demonstrates that chronic continuous hypoxia can be beneficial in preclinical models of mitochondrial disease, auto-immunity, ischemia, and aging. This research is revealing new and unexpected facets of oxygen biology, but translating these findings to patients poses major challenges, as hypoxia can be dangerous. Overcoming these barriers will require integrating insights from high-altitude medicine, sports technology, basic science, and clinical disciplines. Here, we explore the foundations of this nascent field and outline a path to determine how chronic continuous hypoxia can be safely, effectively, and practically delivered to patients.

## One-sentence summary

Though hypoxia is often pathologic, chronic continuous hypoxia has shown promise in many disease models, inspiring a roadmap for clinical translation.

## INTRODUCTION

Human disease stems from the complex interplay of genes and environment. The current era of molecular medicine focuses on repairing the root genetic cause of disease. Nearly two-thirds of drugs approved by the Food and Drug Administration (FDA) in the past decade are supported by human genetic evidence (1), there are 9 FDA-approved gene therapies and over 2,000 trials of gene therapies underway (2). Yet, we cannot forget the second half of the “gene-by-environment” equation. For complex polygenic diseases, the environmental contribution to pathology can exceed that of any individual gene’s contribution. Diet, exercise, ultraviolet (UV) radiation, and pollution are often discussed as key environmental determinants of human health; we propose that the amount of oxygen in ambient air might be a similarly influential environmental parameter.

Supplemental oxygen is a common medical intervention and over 1.5 million Americans are currently prescribed chronic supplemental oxygen (3). Scientists and clinicians have long researched, and in some instances, prescribed exposure to an oxygen concentration different from that of ambient air as a treatment. The United States (US) Navy first used hyperbaric hyperoxia for the treatment of diving sickness in the 1940s (4). At least four therapeutic paradigms premised on modifying normal tissue oxygen tension have been studied in both the laboratory and the clinic for decades: hypoxic preconditioning, remote ischemic preconditioning, acute intermittent hypoxia, and hyperbaric hyperoxia.

Preclinical studies beginning in 2016 revealed the potential benefits of a new oxygen paradigm, chronic continuous hypoxia (CCH). Chronic continuous exposure to between 7% and 11% oxygen at sea level has demonstrated benefit in mouse models of Leigh syndrome (LS) (5–7) and Leigh-like syndrome (8), optic neuropathy (9), Friedreich’s ataxia (FA) (10), multiple sclerosis (11), an accelerated aging phenotype (12), and recovery from myocardial infarction (13) and ischemic stroke (14, 15) (Table 1). In these studies, animals are placed in chambers where hypoxia is achieved by dilution of ambient air with nitrogen. Preclinical studies of CCH have used normobaric hypoxia, in which the oxygen concentration is diminished while remaining at standard atmospheric pressure, as opposed to hypobaric hypoxia, experienced at altitude, in which the oxygen concentration remains 21% but oxygen tension is decreased by lower atmospheric pressure.

We define CCH as at least three days at an oxygen concentration less than 21% at sea level with no more than minimal interruption. Key distinguishing features of CCH and the other four approaches to therapeutically altering normal tissue oxygen tension are summarized in Table 2. We further categorize the degree of CCH as minimal, moderate, or extreme (Table 3). We define oxygen concentrations 17%-20.9% oxygen at sea level as minimal hypoxia, as approximately 500 million people inhabit altitudes with these equivalent oxygen concentrations (16) and lowlanders routinely travel to these altitudes and immediately engage in recreational exertion. We define extreme hypoxia as less than 10% oxygen at sea level, as there are no permanent human settlements above the altitude with this equivalent oxygen concentration and almost all lowlanders require a period of acclimatization or supplemental oxygen to safely visit these altitudes. Even among the small population that is native to an altitude equivalent to 11% oxygen at sea level, it is common to descend to lower elevations to give birth (17). We define moderate hypoxia as between 10%-17% oxygen at sea level. Moderate CCH has shown the most promise for clinical translation, and thus moderate CCH is the focus of this Review.

Recently, a tolerability study was conducted of five healthy volunteers continuously breathing air diluted with nitrogen gas in a hypoxic tent starting at 16% oxygen, decreasing to 11% oxygen over 5 days, and remaining at 11% oxygen for 24 hours; this regimen was safe and well-tolerated (18). Moreover, four patients with recent myocardial infarction have completed a clinical trial of 4.5 days at continuous 12% oxygen at the German Aerospace Center to assess safety and potential to stimulate left ventricular recovery, and this was also safe and well-tolerated (19).

This confluence of promising preclinical and early human studies creates optimism that therapeutic hypoxia could become a reality. Still, many important questions remain. Hypoxia can be dangerous; how can hypoxia be delivered such that its benefits exceed its safety risks? Is it possible to translate hypoxia into the clinic while its full *in vivo* mechanisms are still being elucidated? A sobering aspect of preclinical studies to date is that for CCH to be effective, its delivery needs to be truly continuous without meaningful interruption. Whether this paradigm can be implemented in a practical way remains to be determined.

Here, we review both the opportunities and obstacles involved in making CCH a therapy. First, we review pre-clinical models in which CCH has been showing promise and discuss the purported *in vivo* mechanisms of rescue. Second, we highlight new concepts in oxygen biology that have emerged from preclinical studies of CCH. Third, we provide a rationale for nominating new diseases that might be amenable to CCH. Fourth, we discuss different modalities to achieve therapeutic hypoxia. Fifth, we articulate key barriers that must be considered for clinical translation. Finally, we propose a framework for advancing CCH into clinical trials.

## THE PROMISE OF CCH IN PRECLINICAL MODELS

The *Ndufs4* knockout model of LS was the first mouse model in which therapeutic benefit of CCH was demonstrated (5) (Table 1). In *Ndufs4* knockout mice, CCH with 11% oxygen commenced at the time of weaning (4 weeks of age) extended median lifespan from around 75 days to around 270 days, preserved neurologic function and decreased characteristic brain lesions (5, 6). Further, 11% oxygen commenced after the onset of advanced encephalopathy restored bodyweight and neurologic function, and reversed MRI lesions (6). Hypoxia rescue of *Ndufs4* knockout and a subset of mitochondrial complex I mutants is conserved from mouse to *C. elegans*. *C. elegans* with a homozygous deletion in *lpd-5* (ortholog of *Ndufs4*) were not viable at 21% oxygen but developed to sterile adulthood at 1% oxygen (20). In the context of these whole animal models, hypoxia did not rescue complete loss-of-function mutations in core subunits of complex I. Rather, rescue was restricted to mutations in peripheral subunits, including NDUFS4, composing the soluble mitochondrial matrix arm that transfers electrons from NADH to Coenzyme Q (CoQ). In multiple such mutants, hypoxia restored electron forward flow, thereby restoring complex I activity in a manner dependent upon CoQ binding pocket residues (20).

Although activation of the hypoxia inducible factor (HIF) system can rescue cultured cells from electron transport chain (ETC) toxins (7), the hypoxic rescue of growth phenotypes in *C. elegans* appears largely independent of *hif* (20). Similarly, in *Ndufs4* knockout mice, constitutive HIF activation achieved with *Vhl* or *Phd* mutants was not sufficient for any lifespan extension (7). Moreover, in *lpd-5* null *C. elegans*, overexpression of reactive oxygen species (ROS)-detoxifying enzymes was of no benefit (20). The ability of CCH to rescue primary pathologies of the ETC in mice extends to complex II as well; chronic 10% oxygen commenced prior to induced knockout of the *Sdhc* subunit of complex II more than tripled median survival (8). A growing body of data suggests that perhaps high “unused oxygen” in the context of *Ndufs4* knockout might be the key effector of disease (7), a fairly new concept which is discussed below.

In the sh*Fxn* mouse model of Friedreich’s ataxia (FA), 11% oxygen preserved neurologic function 12 weeks after doxycycline-induced *Fxn* knockdown (10). This rescue is also evolutionarily conserved to *C. elegans*, and here the therapeutic effect of CCH *in vivo* is likely related to the effect of hypoxia *in vitro*; hypoxia activates iron-sulfur cluster biosynthesis in *in vitro* reconstituted systems. In addition, it has long been known that the stability of iron-sulfur clusters is decreased at high oxygen concentrations (21). Hence, hypoxia appears to normalize the quantity of iron-sulfur clusters in the absence of FXN both by boosting their production and promoting their stability (10).

Multiple key features are shared by the *Ndufs4* knockout and sh*Fxn* models. First, hyperoxia (55% oxygen) led to rapid deterioration, and in the case of *Ndufs4* knockout, death within one week (5, 6, 10); the rapidity of this demise is consistent with direct oxygen toxicity. Second, the beneficial effects of hypoxia and detrimental effects of hyperoxia are evolutionarily conserved from mouse to *C. elegans*. Third, CCH was effective not only when started prior to disease manifestations, but it also reversed neurologic dysfunction when commenced in late-stage disease (6, 22). Fourth, although effective at improving central nervous system pathology, CCH did not alleviate cardiac dysfunction in either model. Understanding the basis for this tissue specificity is an ongoing challenge. Accordingly, CCH did not extend lifespan of sh*Fxn* mice, for which cardiomyopathy is the proximate cause of death (10). In *Ndufs4* knockout mice, CCH dramatically extended lifespan, but the mice still succumbed at one year, likely due to cardiac disease (6). Finally, although CCH was protective against neurologic disease, intermittent hypoxia (16 hours 11% oxygen, 8 hours 21% oxygen) was ineffective in *Ndufs4* knockout (6) and even detrimental in sh*Fxn* (22); this is a major practical limitation.

Although CCH does not appear to alleviate cardiac disease in the sh*Fxn* or *Ndufs4* knockout models, a continuous hypoxia regimen which included two weeks at 7% oxygen did promote recovery of the left ventricle after left anterior descending artery ligation in mice (13). In the surviving cardiomyocytes, there was decreased mitochondrial mass and a metabolic shift towards glycolysis, which enabled these surviving cardiomyocytes, which were post-mitotic under normoxia, to engage in mitosis and repopulate the damaged left ventricle with functional cardiomyocytes (13). This was accompanied by increased ejection fraction and capillary density and decreased fibrotic scar (13).

CCH appears to be effective in several disease models characterized by neuro-inflammation. In the *Ndufs4* global knockout model, there was microglial activation (23, 24) secondary to neuronal and glial mitochondrial dysfunction (25), all of which could be prevented (5, 7) or reversed (6) by hypoxia. However, the effect of CCH on neuroinflammation is nuanced. In a retinal ganglion cell-specific *Ndufs4* knockout model of Leber hereditary optic neuropathy, CCH evidently acted upstream of neuroinflammation, since 11% oxygen commenced at weaning led to preservation of retinal ganglion cells and decreased axonal degeneration up to at least 90 days, but did not attenuate the microglial activation that accompanied cell death (9).

While ETC dysfunction in microglia is sufficient to cause neuro-inflammation (26), the effect of CCH is not limited to models with primary mitochondrial dysfunction. In the experimental autoimmune encephalomyelitis (EAE) model of multiple sclerosis and the photothrombotic model of acute cortical stroke, histopathology demonstrated that CCH modulated endothelial cell barrier function, apoptosis of inflammatory leukocytes and monocytes, and microglial activation (11, 14). We have recently shown that CCH extended lifespan and delayed neurologic debility in the *Ercc1 Δ/-* mouse model of accelerated aging characterized by extensive brain and spinal cord microglial activation (27), even though to the best of our knowledge there was no mitochondrial dysfunction present (12). The fact that CCH was effective in so many different models which feature neuro-inflammation anticipates broader mechanisms by which it confers neuronal resilience.

## NEW BIOLOGY OF OXYGEN FROM STUDIES OF CCH

The very idea that hypoxia could be beneficial is counter-intuitive. Perhaps for this reason, CCH is revealing new or under-appreciated concepts in oxygen biology that may help us to understand the origins of certain diseases and the full therapeutic mechanisms of hypoxia.

### A new emphasis on “unused oxygen”

**Figure 1.**
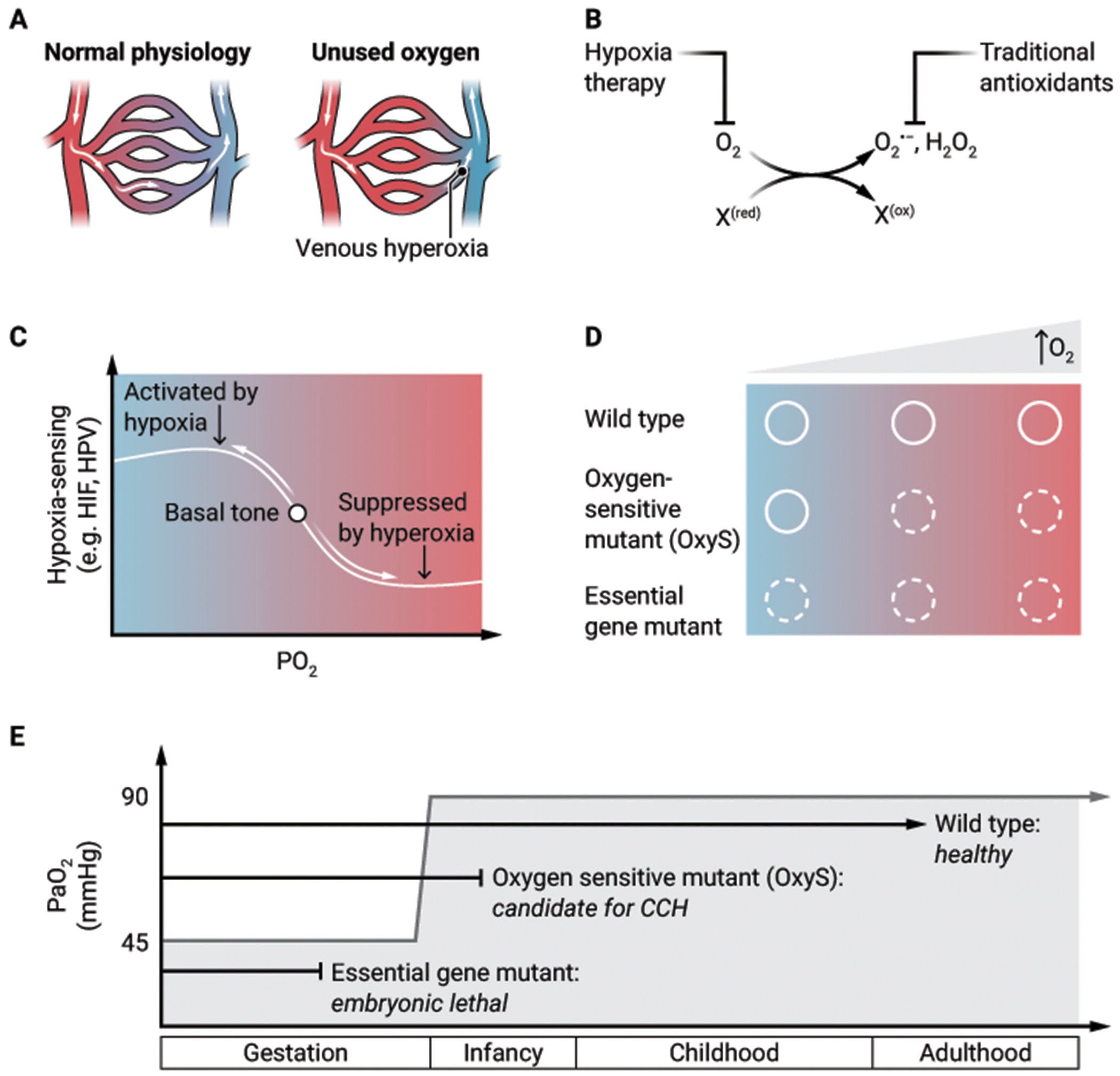
New facets of oxygen biology. **(A)** When arterial oxygen delivery exceeds tissue oxygen consumption, the resulting “unused oxygen” can manifest as venous hyperoxia. **(B)** Oxygen toxicity is classically attributed to partially reduced oxygen species (for example, O_2_^−^, H_2_O_2_) or ROS that are targeted by traditional antioxidants. Hypoxia therapy, however, acts upstream to prevent ROS formation and to prevent damage that emerges from excess oxidation of biomolecules (denoted as “X”) that have reduced the O_2_. **(C)** In healthy states, there is a “basal tone” of hypoxia-sensing programs which can be suppressed in the setting of hyperoxia. “HIF” = hypoxia inducible factor, “HPV” = hypoxic pulmonary vasoconstriction. **(D)** For both wildtype genotypes and essential gene mutants, the phenotypes are independent of oxygen tension (solid circle is healthy phenotype, dotted circle is disease phenotype). By contrast, for OxyS mutants, the disease phenotype is suppressed at low oxygen tension but becomes clinically apparent as oxygen tension increases. **(E)** The developmental fates of wildtype, OxyS, and essential gene mutants are overlaid on a plot of arterial oxygen (PaO_2_) (y-axis) versus life-stage (x-axis). Birth leads to an abrupt transition in PaO2. CREDIT: A. FISHER/SCIENCE TRANSLATIONAL MEDICINE

Clinical medicine focuses on oxygen delivery as the primary determinant of tissue oxygen tension. However, studies of CCH have provided a new appreciation for the role of oxygen consumption in determining the steady state tissue oxygen tension (Fig. 1A). In our efforts to understand the therapeutic effect of CCH in the *Ndufs4* knockout mice, we discovered that the partial pressure of oxygen in the brain of *Ndufs4* knockout mice (measured in the hypothalamus and vestibular nuclei) was 60 mmHg in 21% oxygen, compared to 30 mmHg in wildtype mice (7). Similarly, patients with mitochondrial disease have venous hyperoxia both at rest and with exercise (28–30). Although there are multiple potential explanations for this observation, the most parsimonious is that a dysfunctional ETC consumes an insufficient amount of oxygen. Brain hyperoxia from “unused oxygen” could be a proximal mediator of pathology, a contention supported by the observation that multiple approaches to decreasing brain tissue hyperoxia (ambient hypoxia, severe anemia, increased carboxyhemoglobin) all had therapeutic effect in *Ndufs4* knockout mice (7).

### Oxygen toxicity: it’s not all about ROS

The canonical view of oxygen toxicity has focused on ROS (for example, superoxide, hydrogen peroxide) that are targeted by free radical scavengers (31). Yet, in many of the mouse models (and even in human studies) of disease for which CCH is beneficial, therapy with strong and even catalytic antioxidants has not been effective (32). Furthermore, mouse and worm models of LS and FA are sensitive to high oxygen environments and not rescued by forced expression of ROS-detoxifying enzymes (10, 20). These observations lead to a revised view of oxygen toxicity. We speculate that oxygen toxicity additionally emerges from the direct oxidation of biomolecules (such as iron-sulfur clusters, Fe^2+^, Cu^2+^, persulfides, methionine, certain lipids) by molecular oxygen. In this model, an overlooked source of pathology is the oxidized biomolecule itself which has donated electrons to create the partially reduced oxygen species. Hypoxia might act both by preventing oxidation of labile biomolecules and preventing the formation of ROS, whereas traditional antioxidants only target ROS (Fig. 1B).

### Suppression of the “basal tone” of HIF and other hypoxia sensing programs

The field of oxygen sensing is dominated by the study of HIF signaling (33–35), which is critical to our body’s adaptations to hypoxia (36). HIF is regulated by prolyl hydroxylase domain (PHD) proteins, the canonical example of the 2-oxoglutarate-dependent enzymes, a family of over 60 human dioxygenases encompassing the histone lysine demethylases including the jumonji C domain lysine demethylases and ten-eleven translocation (TET) DNA hydroxylases (37). The oxygen-sensing programs regulated by these enzymes are activated by hypoxia and have important roles in epigenetic regulation (38). Moreover, at the level of whole-organism physiology, hypoxia regulates vascular tone and therefore the distribution of blood flow.

Seldom discussed, however, is that these very hypoxia-sensing programs, including HIF expression (39), have a “basal tone”, which can be suppressed by hyperoxia (40). Several 2-oxoglutarate-dependent enzymes (PHD2, lysine demethylase 5 [KDM5], TET) have had a Michaelis constant (K_m_) for oxygen measured between 30 μM and 100 μM, roughly approximating normal cellular oxygen concentration (41). In the face of ETC inhibition, ambient hypoxia is far less effective at stabilizing HIF, because cellular oxygen tension remains relatively high (42). This raises the intriguing possibility that in states of inherited or acquired ETC dysfunction, CCH might work to restore the “basal tone” of hypoxia-sensing pathways (Fig. 1C). We have shown that *Ndufs4* knockout mice have impaired hypoxic pulmonary vasoconstriction, which was restored by continuously breathing 11% oxygen for three weeks (43), suggesting that hypoxia-sensing systems can be restored by bringing ambient oxygen within its linear range for sensing.

### Oxygen-sensitive mutants

In classical genetics, the notion of temperature-sensitive (Ts) mutants is well appreciated (44). We propose that ambient oxygen ought to be considered similarly to this paradigm. Analogous to genetic screens for Ts mutants, our laboratory performed genome-wide screens that identified hundreds of gene knockouts whose viability was a function of ambient oxygen. We can analogously term them oxygen-sensitive (OxyS) mutants (Fig. 1D), a term that has been applied in bacterial genetics (45) but to our knowledge has not been in employed in mammalian genetics. Of note, 76 of these genes are mutated in human Mendelian diseases (46). Based on animal studies, *NDUFS4* and *FXN* knockouts fulfill the criteria of OxyS mutants. The concepts of Ts and OxyS mutations are important for therapeutic development. For example, the ΔF508 mutation in *CFTR* (which underlies the majority of cases of cystic fibrosis) is a Ts mutant (47), and small molecules that stabilize this protein are highly effective therapies. Analogously, for those monogenic diseases in which CCH exerts its therapeutic benefit through a direct effect on the mutant protein, a small molecule that mimics the mechanism of hypoxia could become a therapy.

### A permissive hypoxic environment *in utero*

The broader question naturally arises: why might OxyS mutants even be compatible with life? We propose that the allelic spectrum of human disease may be shaped by the hypoxic environment *in utero* (Fig. 1E). The human genome has over 3 billion base pairs (48), yet the total number of disease-causing variants likely numbers in the hundreds of thousands (49), presumably because so many mutations are embryonically lethal. Just as Ts mutants can survive at a “permissive” temperature, it is conceivable that *de novo* or inherited OxyS mutations are buffered during embryonic development by the “permissive” hypoxic environment *in utero* (umbilical venous partial pressure of oxygen (pO_2_) is around 45 mmHg, approximately one-half of normal post-natal arterial pO_2_ at sea level (50)), but following birth, become vulnerable to high ambient oxygen. These are the very mutants that could benefit from CCH. Our ontogeny hypothesis is supported by the clinical observation that hyperoxia is particularly detrimental for premature neonates who by developmental age should still be in the hypoxic environment *in utero*; high exposure to supplemental oxygen is one of the leading risk factors for developing retinopathy of prematurity (51).

## DISEASES POTENTIALLY AMENABLE TO CCH THERAPY

### Extrapolation from pre-clinical studies of CCH

Additional diseases treatable with CCH are likely to be found amongst the diseases that share key pathophysiological features with those disease models in which CCH is currently showing benefit (Table 1). Three broad classes are mitochondrial diseases, diseases characterized by neuroinflammation, and recovery from ischemia-reperfusion injury.

To date, more than 300 monogenic mitochondrial disease genes have been identified (52), yet effective therapies are wholly lacking. The genetic heterogeneity and pleiotropy of genetic mitochondrial disorders makes therapeutic development challenging, as does the ultra-rare nature of many individual disorders. For example, there are only a few known cases of patients with mutations in *NDUFS4* or *SDHC*. Moreover, preclinical studies are demonstrating that CCH is beneficial for the central nervous system but not the cardiac manifestations of these disorders. Future clinical trials in genetic mitochondrial disease must therefore be cognizant of both the underlying gene mutation and organ pathology of patients enrolled. Studies in mouse and *C. elegans* models are crucial to guide patient selection in this regard.

A major theme that has emerged from CCH research is its potency in diseases marked by central nervous system inflammation. In the three mouse models with neurologic dysfunction alleviated by CCH, neuroinflammation is a major histopathological feature. It is notable that in *Ndufs4* knockout mice, pharmacologic depletion of leukocytes decreased the burden of characteristic Leigh lesions (23). In the EAE model, CCH accelerated recovery through mechanisms that include both decreased vascular leak and increased apoptosis of injurious leukocytes that accumulated in the spinal cord (11). The brains and spinal cords of *Ercc1 Δ/-* mice demonstrated progressive microglial activation (27), and though it is not yet known if this histopathological finding is ameliorated by CCH, CCH did delay neurologic debility (12). CCH might be beneficial in the large number of neurodegenerative diseases characterized by vicious cycles of neuroinflammation (53).

CCH is also showing beneficial effect in recovery from ischemia-reperfusion injury. In a mouse model of acute myocardial infarction from proximal left anterior descending artery ligation, 7% oxygen achieved gradually and maintained for two weeks decreased mitochondrial mass and oxidative phosphorylation, enabling the surviving cardiomyocytes to proliferate and leading to improved left ventricular ejection fraction and lessened scar tissue (13). In a mouse model of thrombotic cortical stroke, 11% oxygen begun 48 hours after injury and maintained for 2 weeks improved motor function, increased brain-derived neurotrophic factor (BDNF), and limited neuronal loss and microglial activation (14).

Paradoxically, hyperbaric hyperoxia regimens have also demonstrated benefit in rodent models of ischemic stroke (54) and traumatic brain injury (55), in which, like CCH, hyperbaric hyperoxia suppressed inflammation (56) and increase blood vessel formation (55). Determining if the optimal ambient oxygen concentration is biphasic in certain pathologies will require future experiments. It is conceivable that CCH and hyperbaric hyperoxia exert beneficial effects at different timepoints, with the beneficial effect of hyperbaric hyperoxia observed early after vessel occlusion (54) and that of CCH later (14). It is unlikely that hyperbaric hyperoxia is generally helpful for acute non-neurological medical conditions given that liberal targets for arterial oxygen saturation are associated with higher mortality in patients in medical intensive care units (57).

### Epidemiology of high altitude

Epidemiological studies of humans at high altitude can also help to nominate diseases that might be amenable to CCH. Humans inhabit locations with a wide range of oxygen tensions, from sea level to La Rinconada, Peru at 5,100 meters above sea level, with such lower barometric pressure that it is equivalent to 11% oxygen at sea level. It is estimated that 500 million people live above 1,500 m (equivalent to 17.5% oxygen), 81 million above 2,500 m (15.4% oxygen) and 14 million above 3,500 m (13.6% oxygen) (16) (Table 3). Cardiovascular disease in men is the pathology with the strongest evidence for a beneficial effect of high altitude (58, 59). There is also emerging evidence from Bolivia that approximately two miles above sea level is the elevation most enriched for nonagenarians and centenarians (60). One must be cautious in inferring a beneficial effect of ambient hypoxia from an observed effect of altitude because of many confounding factors, including that some highlander populations have undergone genetic selection in ways that are adaptive for high-altitude living (61) and cannot be recapitulated by simply moving a lowlander to high altitude.

History has provided one highly informative “natural experiment” in which thousands of people were nearly randomly assigned to live at high altitude for an extended period and their health outcomes were compared to a highly similar population that remained at sea level. Between 1965 and 1972, 20,000 soldiers of the Indian Army were assigned to serve at 2-to-3-mile elevations along the border with China for 3-year periods, and their health outcomes were compared to 130,700 comrades stationed at sea level (62). There are two salient findings from this study. First, the incidence of conditions linked to poor metabolic health – hypertension (decreased 24%), Type 2 diabetes (decreased 87%, accompanied by a reduction in glucose area under the curve with an oral glucose challenge), ischemic heart disease (decreased 77%) – was lower in those serving at high altitude. Second, the incidence of active pulmonary tuberculosis (TB), a disease that still causes 10.6 million new infections and 1.6 million deaths per year (63), was 80% lower in those serving at high altitude. It has been known for centuries that high altitude is therapeutic against TB (thus the mountainous location of nineteenth century sanatoriums), and to this day, TB incidence, prevalence, and severity is lower at high altitudes (64). The potential for CCH as an adjunctive TB therapy, perhaps by boosting macrophage interferon-gamma production in a HIF-dependent manner at the site of infection (65), merits additional study.

### High-throughput screens

Our laboratory performed a high-throughput screen to identify OxyS mutants, specifically gene knockouts that were selectively essential at 21% oxygen and not 1% oxygen, and vice versa. We identified candidate genes with disease-associated mutations which might be amenable to CCH treatment (46). Using a genome-wide pool of K562 knockout cells targeting approximately 20,000 genes exposed to 1%, 5% or 21% oxygen for 15 days, 213 such genes were identified, heavily enriched in genes encoding mitochondrial proteins, and 76 of which have reported variants causing Mendelian disease (46). The mitochondrial proteins were heavily enriched in those related to the ETC (particularly complex I), iron-sulfur cluster homeostasis, type II fatty acid synthesis, and synthesis of lipoic acid, which subsequent work confirmed was dispensable in hypoxia in cell culture (66). The 24 extra-mitochondrial candidate genes are linked to a great diversity of cellular processes. Genetic screening in cell lines can help to nominate candidate Mendelian diseases for CCH, but based on our experience, we emphasize the need for further validation in whole animals given the key roles of systemic physiology and homeostasis. Future screens that use different cell types and readouts, and whole animal screens with *C. elegans* and *D. melanogaster*, would likely nominate additional candidate Mendelian diseases genes.

## MODALITIES TO ACHIEVE THERAPEUTIC HYPOXIA

A major challenge for clinical translation of CCH is that preclinical models have used chronic continuous inhalational regimens of less than or equal to 11% oxygen without interruption. In genetic disease models, once the animals are returned to normoxia, the disease returns. Unfortunately, emerging evidence from preclinical models suggests that less intensive regimens might be ineffective, and intermittent regimens that cycle each day (such as 16 hours of 11% oxygen/ 8 hours of 21% oxygen) might be not only ineffective but harmful, likely attributable to the erythropoietic response (22). Preclinical models of CCH have focused on “inhalational hypoxia” (decreased oxygen content in ambient air) as the means to achieve tissue hypoxia. Here we explore some possible regimens for achieving therapeutic hypoxia, which could be used in isolation or in combination.

### Ambulatory facemasks and home oxygen conditioners

Inhalational hypoxia and the closely related concept of scrubbing oxygen from ambient air are found in existing consumer and industrial technologies. These technologies could be repurposed to create a hypoxic home environment and to allow for continuous inhalational hypoxia while outside the home. For example, some competitive endurance athletes have long used the “live high, train low” method to improve performance, sleeping at sea level in “altitude tents” that provide a hypoxic environment by diluting ambient air with nitrogen gas (67). Moreover, portable “altitude generators” that filter oxygen in ambient air to achieve oxygen concentrations as low as 9% enable endurance athletes to “train high”. These consumer devices are commercially available now, though advancements in their miniaturization would greatly facilitate their chronic use by patients. Ultimately, we envision that in the same way home air conditioners today regulate temperature and humidity, the air conditioner of the future could also regulate oxygen in the home.

### Moving to high altitude

With 14 million people worldwide permanently inhabiting elevations above 3,500 m (16) (equivalent to 13.6% oxygen at sea level), there are multiple locations to which a patient might move to access “natural” CCH. Permanent settlements above 3,500 m are found in Argentina, Bolivia, Bhutan, Chile, China, India, Kyrgyzstan, Nepal, Peru and Tajikistan. Although many of these high-altitude settlements are small villages in which medical infrastructure to care for patients with complex disease might need to be built, the list includes major cities as well. El Alto, Peru (elevation 4,150 m, equivalent to 12.5% oxygen at sea level) has a population of approximately 1 million people, suggesting that it could host patients without overwhelming local infrastructure.

### “Hypoxia in a pill” using small molecules

An orally available small molecule regimen would be most welcome. Tissue oxygen can be decreased by increasing hemoglobin affinity for oxygen (left-shifting) such that at any given arterial oxygen tension, less oxygen is unloaded into the tissues. Two long-known modifications of hemoglobin, carboxyhemoglobin and methemoglobin, are potently left-shifted. Carboxyhemoglobin up to 14% is considered safe enough (based on concentrations observed in heavy smokers) that carbon monoxide has been tested in over 25 clinical trials for a variety of conditions (68). Efforts to pharmacologically left-shift hemoglobin have advanced in the past decade as part of sickle cell disease (SCD) research. First, because fetal hemoglobin does not have the sickle mutation, strategies to increase fetal hemoglobin have been developed (the partial pressure of oxygen at which hemoglobin is 50% saturated, p50, is 19 mmHg for fetal hemoglobin versus 27 mmHg for adult hemoglobin). Second, because Hemoglobin S sickles in the deoxyhemoglobin state but not in the oxyhemoglobin state, strategies that preferentially maintain the oxyhemoglobin state mitigate vaso-occlusion and hemolysis (69). Two hemoglobin left-shifting drugs have been approved by the FDA, voxelotor (previously GBT440) (70) for SCD and mitapivat (71, 72) for hemolytic anemia.

Our laboratory recently reported preclinical proof-of-concept for “hypoxia in a pill” in *Ndufs4* knockout mice. By increasing hemoglobin affinity for oxygen with GBT440 and simultaneously blunting the compensatory erythropoietic response with the HIF-2α inhibitor PT2399 (approved for renal cancer), brain tissue oxygen tension in wildtype mice decreased 36%, of similar magnitude to the effect of breathing continuous 11% oxygen (73). The addition of the HIF-2α inhibitor was essential for achieving brain tissue hypoxia, because the increased hemoglobin that resulted from GBT440 alone increased brain oxygen tension. In *Ndufs4* knockout mice, this drug combination increased median and maximal lifespan and locomotor function and decreased brain lesions on MRI (73). The effect size of “hypoxia in a pill” on lifespan was not as large as continuously breathing 11% oxygen, perhaps because of the short half-life of GBT440 and the fact that the drugs were only given five days per week for practical reasons. We note that GBT440 remains FDA-approved but was recently withdrawn from the market because of safety concerns in SCD patients, the details of which are not yet reported. Regardless, this work demonstrates that “hypoxia in a pill” is pharmacologically tractable.

### Procedures to decrease arterial oxygen content

For completeness, we consider medical or surgical procedures for achieving therapeutic hypoxia. Therapeutic phlebotomy is a useful tool in the modern treatment of conditions of iron overload including hemochromatosis, polycythemia vera and porphyria cutanea tarda (74), where long-term removal of up to 300 ml of blood monthly resulting in a hemoglobin around 10 g/dL has been well-tolerated (75). Another physical intervention to decrease the oxygen content of arterial blood is the purposeful creation of an anatomic shunt allowing deoxygenated blood to pass from the right side of the heart to the systemic circulation without becoming oxygenated in the lungs. Given that severe congenital heart defects resulting in hypoxemia require surgical correction, the notion of intentionally creating a cardiac shunt at first sounds as counter-intuitive as therapeutic hypoxia itself, but in fact, there is precedent for this approach. The intentional creation of an 8 mm atrial septal defect is under investigation for the treatment of heart failure with preserved ejection fraction (76) (a condition in which decreased peripheral oxygen extraction during exercise is common (77)). Neither phlebotomy nor shunt creation could feasibly be the primary means of safely achieving tissue hypoxia, but their use could serve as an adjunct and to blunt compensatory responses.

## KEY BARRIERS FOR THE CLINICAL TRANSLATION OF CCH

### Extrapolating from mice to humans

Extrapolating from mice to humans is always challenging and differences between mouse and human oxygen physiology merit special consideration. First, differences in basal metabolism complicate directly translating specific CCH regimens from mice to humans. Resting oxygen consumption per bodyweight is approximately five times higher in mice (78, 79). Second, the mouse heart has less reserve capacity to increase cardiac output (80). Third, under resting conditions, mice distribute only 3% of cardiac output to the brain (81), as opposed to around 15% in humans. Fourth, though there is a paucity of high-quality direct measurements of tissue oxygen tension in all species (and especially in humans), available data suggests that human baseline brain tissue oxygen tension is modestly higher than that of rodents (82). Fifth, human hemoglobin has greater oxygen affinity than mouse hemoglobin (p50 human: around 27 mmHg, p50 mouse: around 45 mmHg) (83). Because of these fundamental differences in oxygen physiology, the “dose” of hypoxia required in humans will very likely differ from that in mice; it could be higher or lower, and cannot be easily predicted. Furthermore, patients have a greater diversity of these key parameters in oxygen metabolism than do laboratory mice, so the optimal regimen might require personalization for an individual patient.

### Commencing therapy in young versus old

Mouse studies also offer a cautionary note that the timing of CCH might determine its overall benefit-risk ratio. Although CCH commenced at 4 weeks of age extended lifespan in rapidly aged mice (12), chronic continuous 11% oxygen accelerated death in wildtype C57BL/6 mice when started at 21 months of age, because of pulmonary hypertension and right ventricular failure (84). Analogously, whereas caloric restriction has extended lifespan in multiple species, when commenced after two years of age it hastened mortality in mice (85, 86). To date, CCH has proven beneficial in mice under one year of age. It is conceivable that the benefits of CCH are limited to younger organisms. It might be more difficult to safely achieve decreased tissue oxygen tension in older organisms, as their resting oxygen use decreases. Whole body resting oxygen utilization is decreased 19% in 24 month old mice compared to 12 month old mice (87), and similarly decreases as humans age (88). Moreover, whether reversal of neuropathology could be achieved in mice older than one year, who recover from neurologic injury more slowly than young mice (89), remains to be tested.

### Measuring tissue oxygen tension

Clinical development of CCH would benefit from new tools to measure tissue oxygen tension. Even in mice, measuring brain oxygen tension with invasive probes is complicated by spatial heterogeneity with small alterations in capillary blood flow causing spontaneous formation of “hypoxic pockets” (90). Of course, in humans, such direct measurements are rarely feasible or clinically justified. Several non-invasive methods, such as BOLD MRI with phase contrast imaging (91), PET in conjunction with the inhalation of ^15^O-labeled molecular oxygen (92), and cerebral oximetry by near-infrared spectroscopy (93) offer correlates of brain tissue oxygen tension, but each has its limitations. Cerebral oximetry by near-infrared spectroscopy utilizes the same principle as arterial oxygen saturation (“finger”) probes, detecting the differential absorption of near-infrared light by oxygenated versus deoxygenated hemoglobin. Though imperfect, cerebral hemoglobin saturation measured by near-infrared spectroscopy is sufficiently correlated with brain tissue oxygen tension that it can reliably identify brain tissue hypoxia from a known normoxic baseline (94). Because of its convenience and ability for continuous recording, it is already in widespread use in anesthesia monitoring (93) and has guided interventions in clinical trials seeking to optimize brain oxygenation (95, 96). While we are optimistic about cerebral oximetry as a tool in CCH clinical trials, a non-invasive method to quantitatively measure oxygen tension at the cellular level *in vivo* would be a welcome technological advance.

### Acute and long-term hazards of hypoxia

**Figure 2.**
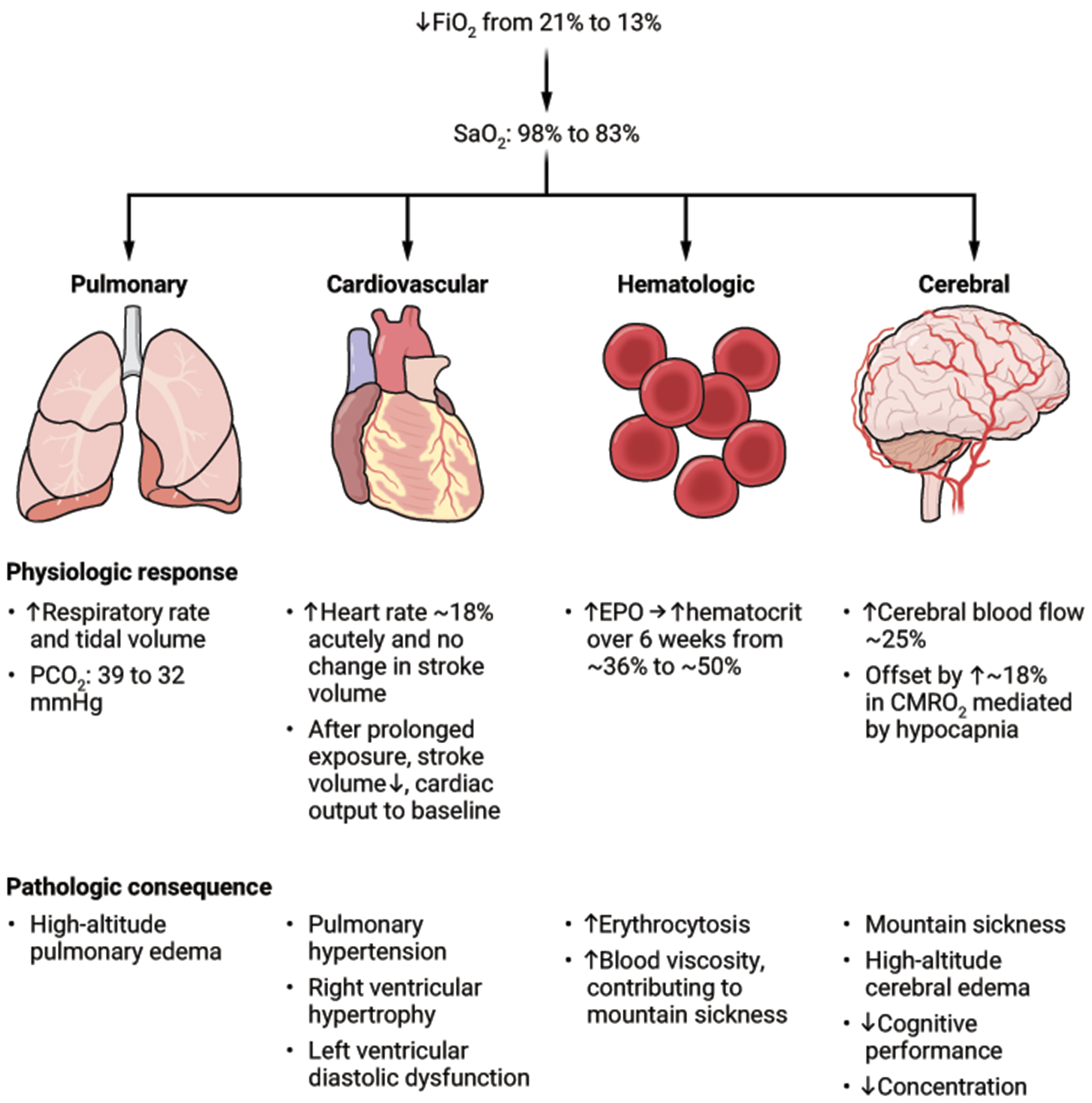
Physiological responses to and pathological consequences of hypoxia for different organ systems. Quantitative estimates are based on studies of healthy adult volunteers in the first 48 hours after moving from sea level to a 13% normobaric hypoxia chamber or the altitude equivalent to this oxygen concentration (hypobaric hypoxia). “CMRO_2_” = cerebral metabolic rate of oxygen consumption (107–110). CREDIT: A. FISHER/SCIENCE TRANSLATIONAL MEDICINE

The safety concerns with CCH necessitate carefully identifying the populations in whom potential benefits outweigh risks (Fig. 2). The first safety concern of CCH is the acute effect of hypoxia, which ranges in severity from acute mountain sickness (headache, nausea, lethargy) to the potentially lethal complications of high-altitude pulmonary and cerebral edema (97). These acute effects of hypoxia can generally be mitigated by gradual acclimatization. However, many lowlanders who move to high altitude experience chronic mountain sickness (97) and small but measurable decrements in cognitive performance even after a year (98). Chronic hypoxia-mediated vasoconstriction of the pulmonary arterial vasculature causes pulmonary hypertension and signs of right heart failure in at least 5% of those who permanently reside at altitudes above 3200 m (equivalent to around 14.1% oxygen at sea level), though the proportion who are symptomatic from this physiologic abnormality is not known (99, 100). There is higher mortality for individuals with chronic obstructive pulmonary disease who reside at higher altitudes (59); CCH is unlikely to be tolerated in those with hypoxemic lung disease. Certain rare pathologies are seen at much higher rates in lifelong highlanders, such as carotid body tumors (101), a neuroendocrine neoplasm that is exceedingly rare in lowlanders. Consideration of CCH as long-term therapy should be reserved for those conditions in which the gravity of the prognosis and the paucity of treatment options justify these risks.

### A barrier to efficacy: homeostasis

Delivery of inhalational hypoxia does not guarantee tissue hypoxia, particularly brain tissue hypoxia, because humans have robust homeostatic mechanisms to defend brain tissue oxygenation. In a group of 17 trekkers in the Nepal Himalayas, cerebral hemoglobin oxygen saturation measured using near-infrared spectroscopy decreased modestly from 65% at sea level to 60% at an altitude of 3450 m (equivalent to around 13.7% oxygen at sea level); only at altitudes above 4400 m (equivalent to around 12.1% oxygen at sea level) was a marked drop to about 50% observed (102). In healthy volunteers who ascended to 3700 m (equivalent to around 13.2% oxygen at sea level) from sea level in 6 hours, resting cerebral hemoglobin oxygen saturation was the same as at sea level and required exercise to decrease (and then increased above resting saturation during post-exercise recovery) (103). In a study of 44 critically-ill children requiring air transport in cabins pressurized to the equivalent of 1 mile above sea level (equivalent to around 17% oxygen at sea level), there were highly variable effects on cerebral hemoglobin oxygen saturation, with only 56% of children demonstrating a decrease (104). Underscoring the capacity for chronic adaptation to ambient hypoxia, studies in native Kyrgyzstan highlanders at 3250 m (equivalent to around 14% oxygen at sea level) found no difference in cerebral hemoglobin oxygen saturation compared to healthy lowlanders (105).

These studies suggest that a reliable decrement in brain oxygenation does not occur until altitudes of around 4000 m (equivalent to around 12.7% oxygen at sea level) (102, 104, 106). This observation accords with the physiological compensations that have been noted in healthy volunteers upon exposure to the equivalent of around 13% oxygen at sea level (Fig. 2), which include acute hyperventilation (approximate 20% decrease in alveolar partial pressure of carbon dioxide causing increased partial pressure of oxygen in the alveolus) (107, 108), tachycardia-mediated increase in cardiac output (around 18%, which increases systemic oxygen delivery) (109), increased cerebral blood flow (by approximately 25%) (107, 108), and starting within 48 hours but requiring six weeks to reach full effect, increased erythropoiesis to increase hematocrit (to approximately 50%) (110). Indeed, the hematocrit response to altitude is very sensitive, with increases in mean hematocrit relative to sea level observed at elevations of 1000 m (111).

For patients with brain hyperoxia in whom the goal is to achieve normal brain oxygen tension, considering homeostatic responses is essential for the clinical translation of CCH. Perhaps these compensatory responses will be less of a hurdle in patients with mitochondrial disease than in healthy volunteers. Mice with ETC dysfunction have impaired physiologic responses to hypoxia such as hypoxic ventilatory drive (112), hypoxic pulmonary vasoconstriction (43) and the hypoxic erythropoietic response (113), but we await data on the physiologic responses to CCH of patients with mitochondrial disease.

### Duration and durability of CCH therapy

The required duration of hypoxia is likely to vary greatly depending on the indication. For recovery from ischemic insults, after which there is a defined window for maximal recovery (114), weeks to months will likely suffice. In the mouse model of recovery from acute myocardial infarction, two weeks of CCH was highly beneficial whereas three weeks increased mortality (13). By contrast, for monogenic diseases, the presumption is that the need for CCH would be life-long, although there is the hopeful possibility that in certain diseases with childhood onset, such as LS, substantial benefit could be attained if CCH was provided through key developmental milestones during which episodic decompensations are particularly devastating (115).

The maximal allowable interruption from hypoxia without loss of efficacy or gain of harm is not yet known. In the *Ndufs4* knockout model, a regimen of 10 hours daily of 11% oxygen/14 hours 21% oxygen was ineffective (6). Crucially, in the sh*Fxn* model, a regimen of 16 hours daily of 11% oxygen/8 hours 21% oxygen was not only ineffective, but harmful relative to full-time normoxia, as the daily “pulse” of normoxia combined with the erythropoietic response led to detrimental periods of hyperoxia. The toxicity of intermittent hypoxia could be prevented by blunting the erythropoietic response with a HIF2α inhibitor (22). Briefer interruptions, such as two to four hours daily, have not been formally tested, though in practice, mice were removed from hypoxia almost daily for up to an hour for cage cleaning, bodyweight measurement, and phenotyping, and this had no obvious deleterious effect. Just as with inter-species differences in the optimal “dose” of hypoxia, there is no proven way to predict whether patients would be more or less sensitive than mice to interruptions in hypoxia.

## PAVING A PATH FORWARD

The key barriers discussed above are specific to CCH, but analogous challenges are posed by all new treatments. Ultimately, the purpose of a clinical trial is to test a hypothesis in humans, and there is inherent uncertainty at the outset. As with any other new therapy, clinical development of CCH can proceed under conditions of uncertainty provided there is rigorous consideration for safety and efficacy at all stages. In designing an initial clinical trial for CCH, there are many possible diseases from which to choose. CCH is showing promise in preclinical models of both common diseases and rare genetic diseases. The common diseases provide a larger pool of eligible patients and some (for example flares of multiple sclerosis, recovery from acute myocardial infarction) may lend themselves to a shorter duration of therapy. Rare monogenic diseases present the highest unmet need. Amongst rare diseases, genetic mitochondrial disorders stand out and LS is of particular interest (Table 1), but a disadvantage is its tremendous genetic, allelic and phenotypic heterogeneity, with more than 113 different underlying genes (116) and multi-systemic manifestations without well-defined natural history. Furthermore, it is a pediatric disease which presents special challenges. Alternatively, FA has higher global prevalence (around 1 in 40,000) (117) with natural history well-characterized (118), typical onset in the teenage years, and a validated metric of disease severity (119) that has served as an approval endpoint (120, 121).

### Defining study populations and objectives for first-in-human CCH trials

Most new therapies are first tested in healthy volunteers to establish their safety, tolerability, and pharmacokinetics (Phase 1) before trials with patients. However, for certain therapies such as gene therapy and cytotoxic chemotherapy, it is unethical or impractical to enroll healthy volunteers, and first-in-human trials begin in patients. Phase 1 studies in healthy volunteers generally consist of giving ascending doses of an investigational product to successive cohorts, carefully monitoring for adverse events, until an “exposure cap” is reached. The long history of physiology studies of healthy volunteers trekking at altitude (102), the known epidemiology of permanent high altitude residents (16) and the recent conclusion of pilot studies with subjects breathing less than or equal to 12% oxygen for days (18, 19) provide much of the knowledge that would be gained from Phase 1 studies of inhalational hypoxia in healthy volunteers. Thus, for inhalational hypoxia (or moving to high altitude) it may be permissible to move directly into patients. For a new small molecule that induces moderate tissue hypoxia, it might also be advisable for first-in-human studies to be conducted in patients who have demonstrated tissue hyperoxia in order to avoid exposing healthy volunteers to the potential risks of tissue hypoxia. Ultimately, the appropriate first-in-human population must be determined by the drug’s specific mechanism of action and preclinical toxicology profile. Clinical trials of invasive procedures are generally only performed in patients.

The first priority in a Phase 1 trial of CCH will be to determine the “maximal dose” at which CCH is safe and well-tolerated. For inhalational hypoxia, a Phase 1 trial should consist of gradually decreasing ambient oxygen (analogous to ascending dose studies) while carefully monitoring for adverse effects with an “exposure cap” of 11% oxygen at sea level, the minimal oxygen tension permanently inhabited by humans. The key safety endpoints to assess during CCH trials are inspired by decades of research in high altitude medicine. Trialists must be cognizant of the known symptoms and signs of altitude illness, particularly those related to cardiopulmonary and neurocognitive function. The brisk response of erythropoietin (EPO) to hypoxia (18) and the sustained response of increased hemoglobin to hypoxia (110) provide readily available “pharmacodynamic” biomarkers to assess that CCH has engaged its therapeutic target. It will also be helpful to have a proxy for oxygen tension in the target tissue; for brain disease, cerebral oximetry by near-infrared spectroscopy could be useful (93).

### Clinical trial duration, location and post-trial care

The Wilderness Medical Society provides guidelines on the rate of ascent that mitigates the risk of acute altitude illness in healthy trekkers, recommending at least 48 hours to ascend 2,750 m and then no more than 500 m every 24 hours (122). With safety as the highest priority, the decrease in ambient oxygen should occur more gradually in patients. Once the maximal safe dose of hypoxia is established in a patient population, it can be tested on a relevant biomarker of disease (Phase 2) and then in a pivotal study assessing actual clinical outcomes (Phase 3). Particularly for rare diseases, it is increasingly common to have a single trial that combines two or more phases (for example, Phase1/2 or Phase 2/3). This trial design could be particularly attractive for CCH as it enables patients who are benefitting from CCH to move seamlessly into the next phase of a trial without interruption. The risks in patients of reverting to normoxia after sustained CCH are unknown, and there is an ethical obligation to ensure continued access to a therapy that has benefited an individual patient. Accordingly, when initiating clinical trials of CCH, accommodations must be made for the option of sustained residence at high-altitude after the clinical trial (at least until such time that fully oxygen-conditioned homes at sea level are a reality). Ensuring continued access to treatments from which a trial participant has benefitted is a common practice in clinical development, especially for rare diseases, where the cost of medications can be very high.

The duration of later-phase trials will depend on the natural history of the disease in question. For genetic mitochondrial diseases, mouse studies predict that objective measures of neurological performance could improve in weeks (6, 10). Moreover, T2-intense MRI lesions in these mouse models disappear after several weeks of hypoxia therapy (6, 7), and there is growing appreciation that these lesions can be reversible in humans (123). Hence it is conceivable that benefits of CCH could be observed within a few weeks. However, we should be prepared to conduct clinical trials that last longer than this, as the time to potential effect in humans remains unknown. In the pivotal trial of omaveloxolone for FA that led to its approval, patients were treated for 48 weeks (120). Because remaining in an enclosed environment for an extended period is unappealing, we believe that for trials longer than 2 weeks moving to altitude may offer an appealing alternative as patients can resume some of their usual daily activities.

### OUTLOOK

We are currently within the first decade of CCH research, and a growing body of preclinical studies are delineating both rare and common diseases that can benefit. As efforts are made to usher CCH into the clinic, we can expect to discover more unanticipated facets of oxygen biology. We are optimistic that new technologies for delivering hypoxia and measuring tissue oxygen tension will be developed and will have widespread uses in clinical medicine. If CCH is proven safe and efficacious for any disease, this will facilitate its development for other diseases. Further challenges will arise in providing for lifelong maintenance of CCH in patients’ native locations, which will require the refinement of hypoxic tent technology, the miniaturization of portable devices, and creative adaptations of existing technologies to support other modalities. Still, it would represent great progress if overcoming these pragmatic challenges were what stood in the way of effective treatment for many devastating conditions for which CCH has shown preclinical promise. The first TB sanatorium at high altitude was founded in 1889 (124), whereas it was not until the 1940s that the first effective antibiotic for TB was manufactured (125). Although the rapid pace of new therapeutic development for rare disease makes us hopeful that we need not wait a comparably long period for effective pharmacologic or gene therapy for genetic mitochondrial disease, CCH could be an important bridge until the time when such therapies are available. We should investigate this possibility while maintaining the highest standards of rigor for safety and efficacy.

## Figures and Tables

**Table 1.** Mouse models with therapeutic effect of chronic continuous hypoxia. The table details different diseases and corresponding mouse models.

Disease	Mouse model	Hypoxia Initiation	Hypoxia Duration	FiO2 (%)	Outcomes	Contributing mechanisms
Leigh syndrome (5–7, 20)	*Ndufs4* knockout	Prevention: age 4 weeks; reversal: age 8 weeks	Lifelong	11%	Increased median lifespan 11 weeks to 39 weeks, improved motor function and decreased brain lesions; reversed advanced neuropathology with recovery of motor function, weight and lifespan extension	Restored complex I activity by increasing electron forward flow; modest increase in protein levels of complex I subunits; attenuated brain hyperoxia
Leigh-like syndrome (8)	*Sdhc* knockout	Age 25 days, 5 days prior to doxycycline treatment to induce *Sdhc* knockout	Lifelong	10%	Increased median lifespan from approximately 35 days post *Sdhc* knockout to greater than 120 days	Suppressed adverse metabolic consequences of decreased complex II activity through currently unknown mechanisms
Friedreich’s ataxia (10, 22)	sh*Fxn*	Simultaneous with doxycycline treatment to induce *Fxn* knockdown	Lifelong	11%	Improved motor function at 12 weeks after FXN knockdown; no effect on mortality from cardiomyopathy	Restored iron-sulfur cluster levels and activity
Multiple sclerosis (11)	Experimental autoimmune encephalo-myelitis	10 to 11 days after immunization with myelin antigen	24 days	10%	Accelerated resolution of limb paralysis	Increased vascular integrity and expression of tight junctions, increased apoptosis of inflammatory leukocytes in spinal cord
Acute myocardial infarction (13)	Left anterior descending artery ligation	1 week after myocardial infarction	Decreased from 21% to 7% over 2 weeks, then maintained at 7% for 2 weeks, then returned to 21% over 1 week	7%	Increased ejection fraction and decreased fibrotic scar area	Proliferation of surviving cardiomyocytes mediated by metabolic shift to glycolysis, decreased ROS-mediated DNA damage and increased capillary size
Ischemic stroke (14, 15)	Thrombotic cortical stroke	2 days after photo-thrombosis causing stroke	2 weeks	11%	Improved motor function	Decreased neuronal death and microglial activation, increased BDNF and capillary density
Accelerated aging (12)	*Ercc1 Δ/-*	Age 4 weeks	Lifelong	11%	Increased median lifespan 16 weeks to 24 weeks with improved motor function	Neuronal resilience through currently unknown mechanisms

**Table 2.** Broad categories of oxygen therapy. The table shows categories of oxygen therapy and details of its use.

Regimen	Oxygen concentration	Duration and Timing	Continuous or Cyclic	Prevention or Treatment	Examples of Preclinical Models	Clinically Tested or Approved* Applications
Acute Intermittent Hypoxia	Approximately 9% to 16% (126)	Repetitive cycles alternating minutes of hypoxia and normoxia up to several hours a day for days to weeks (126)	Cyclic	Treatment	skeletal muscle (127) and respiratory muscle function (128) in spinal cord injury	Recovery of motor function in spinal cord injury (129) ; hypertension associated with obstructive sleep apnea (130)
Hypoxic Pre-conditioning	Approximately 8% to 12% (131)	A few daily sessions consisting of either a few hours of continuous hypoxia or repetitive cycles alternating hypoxia with normoxia/hyperoxia hours to a few days before injury (131)	Continuous or Cyclic	Prevention	Cardiac (131), cerebral (132), renal (133) ischemia-reperfusion injury	Prevention of myocardial injury prior to coronary artery bypass grafting (134)
Remote Ischemic Pre-conditioning	Protection of target tissue such as myocardium by decreased blood flow and subsequent tissue hypoxia of another organ (usually limb)	Approximately 10 to 20 minutes, hours to a few days before injury (135)	Continuous or Cyclic	Prevention	cardiac ischemia-reperfusion injury (135)	Prevention of myocardial injury prior to complex cardiac surgery (136)
Hyperbaric Hyperoxia	100%	Daily sessions consisting of 1 to 2 hours for up to several weeks	Mostly Continuous	Treatment	Stroke (54); traumatic brain injury (55)	13 FDA-approved conditions including carbon monoxide poisoning, wound healing, delayed radiation injury, deep muscle infection (4)
Chronic Continuous Hypoxia	Moderate (10% to 17%); Extreme (less than 10%)	At least 3 days to lifelong	Continuous	Treatment	Leigh and Leigh-like syndromes, Friedreich’s ataxia, experimental autoimmune encephalo-myelitis, stroke recovery, myocardial infarction recovery, accelerated aging	Recovery from acute myocardial infarction (19)

* Only hyperbaric hyperoxia has FDA-approved indications

**Table 3.** Altitude and effective oxygen concentration Atmospheric pressures were calculated from altitude according to the barometric formula as provided by U.S. Standard Atmosphere, 1976 (137). It is known that this formula slightly underestimates the atmospheric pressure at the summit of Everest, the directly measured value of which is reported (138).

Location	Elevation (m)	Effective oxygen concentration	Category of Hypoxia
Sea Level	0	20.9%	Normoxia
Denver, Colorado (USA)	1,609	17.2%	Minimal
Aspen, Colorado (USA)	2,438	15.6%	Moderate
La Paz, Bolivia	3,640	13.4%	Moderate
Mont Blanc Summit	4,808	11.5%	Moderate
Everest North Base Camp	5,150	10.9%	Moderate
Mount Kilimanjaro Summit	5,895	9.9%	Extreme
Everest Summit	8,848	6.9%	Extreme
